# Coulomb Interactions between Cytoplasmic Electric Fields and Phosphorylated Messenger Proteins Optimize Information Flow in Cells

**DOI:** 10.1371/journal.pone.0012084

**Published:** 2010-08-11

**Authors:** Robert A. Gatenby, B. Roy Frieden

**Affiliations:** 1 Departments of Radiology and Integrated Mathematical Oncology, Moffitt Cancer Center, Tampa, Florida, United States of America; 2 College of Optical Sciences, University of Arizona, Tucson, Arizona, United States of America; Tel Aviv University, Israel

## Abstract

**Background:**

Normal cell function requires timely and accurate transmission of information from receptors on the cell membrane (CM) to the nucleus. Movement of messenger proteins in the cytoplasm is thought to be dependent on random walk. However, Brownian motion will disperse messenger proteins throughout the cytosol resulting in slow and highly variable transit times. We propose that a critical component of information transfer is an intracellular electric field generated by distribution of charge on the nuclear membrane (NM). While the latter has been demonstrated experimentally for decades, the role of the consequent electric field has been assumed to be minimal due to a Debye length of about 1 nanometer that results from screening by intracellular Cl^−^ and K^+^. We propose inclusion of these inorganic ions in the Debye-Huckel equation is incorrect because nuclear pores allow transit through the membrane at a rate far faster than the time to thermodynamic equilibrium. In our model, only the charged, mobile messenger proteins contribute to the Debye length.

**Findings:**

Using this revised model and published data, we estimate the NM possesses a Debye-Huckel length of a few microns and find this is consistent with recent measurement using intracellular nano-voltmeters. We demonstrate the field will accelerate isolated messenger proteins toward the nucleus through Coulomb interactions with negative charges added by phosphorylation. We calculate transit times as short as 0.01 sec. When large numbers of phosphorylated messenger proteins are generated by increasing concentrations of extracellular ligands, we demonstrate they generate a self-screening environment that regionally attenuates the cytoplasmic field, slowing movement but permitting greater cross talk among pathways. Preliminary experimental results with phosphorylated RAF are consistent with model predictions.

**Conclusion:**

This work demonstrates that previously unrecognized Coulomb interactions between phosphorylated messenger proteins and intracellular electric fields will optimize information transfer from the CM to the NM in cells.

## Introduction

**Figure 1:**
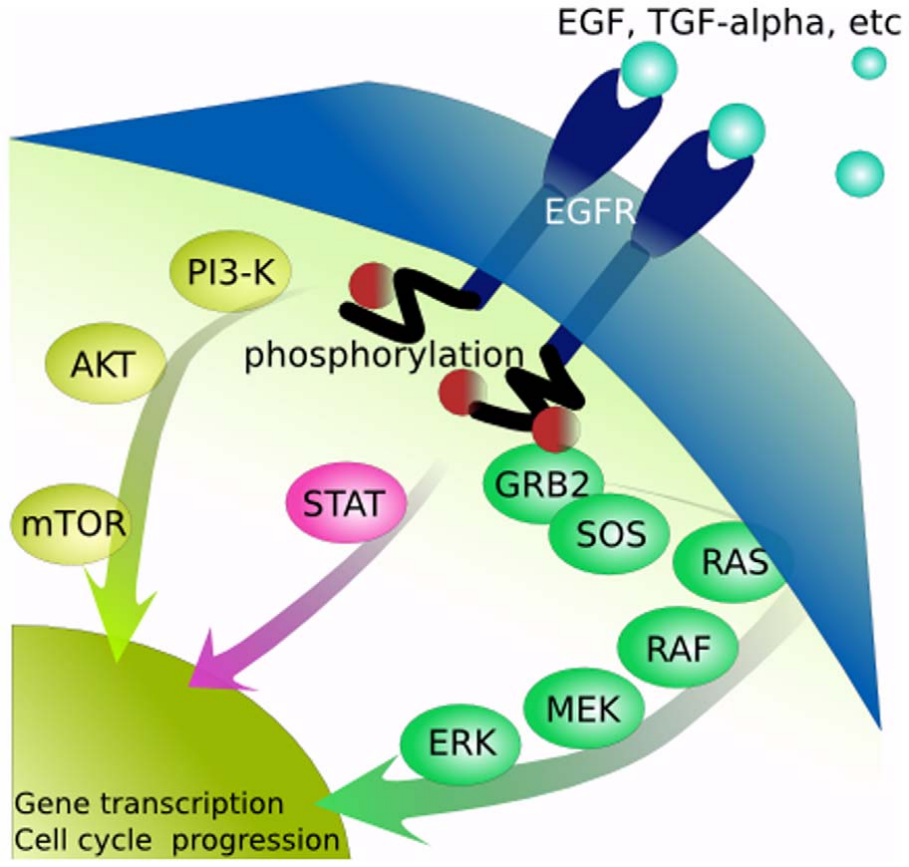
Conventional illustration of the EGFR pathway. The proteins are not drawn to scale and, as a result, the limitation of random walk in allowing rapid and reliable transmission of information by random walk is underestimated. In fact, the distance from the CM to the NM is about 1,000 protein diameters.

The critical role of information in living systems has been well recognized [1]–[4]. Accurate and timely flow of information through messenger proteins, often in multi-protein complexes, from the cell membrane (CM) to the nucleus is necessary for normal function [5]. Extensive investigation has identified the components of intracellular pathways that transmit information from receptors in the cell membrane to the nucleus. The dynamics of the protein-protein interactions have been modeled and there is a large literature on biological information networks [6], [7]. However, these models are rarely spatially explicit and movement of messenger proteins, if considered at all, is assumed to be via diffusion. Fig. 1, for example, is a classic depiction of the EGFR pathway. However, the length scale on Fig. 1 is misleading since a typical protein is about 3 nm in diameter, while the distance from the CM to the NM is generally 3 to 4 µm – about 1,000 protein diameters.

Here we point out that movement of messenger proteins by random walk over a distance between the CM and NM would result in broad dispersal of information in the cytoplasm. This produces slow and highly variable transit times in any cohort of signaling proteins that simultaneously leave the cell membrane. Furthermore, since the phosphorylated messenger proteins are subject to inactivation by phosphorylases within the cytoplasm, dispersal in the cytosol may result in significant information loss. We propose that, because of the limitations of random walk, optimal information transfer from the CM to the NM requires that the messenger proteins undergo *directed motion* toward the nucleus.

Here we examine the movement of messenger proteins as individuals or in small clusters with scaffolding proteins from the CM to the NM. We propose that messenger protein movement is governed by:

1. An intra-cytoplasmic electric field *E*(*r*) generated by the nuclear membrane. We calculate using published data on charge density within the NM, the characteristics of this field and find these predictions are consistent with recent measurement using intracellular nanovoltmeters.
2. Coulomb interactions between the intracytoplasmic electric field and negative charges placed on messenger proteins by phosphorylation. This accelerates the proteins, either as single molecules or clustered multi-protein complexes, toward the nucleus allowing directed motion. While some investigators have speculated that messenger protein movement is facilitated by interactions microtubules and microfilaments, we note published observations seem consistent free movement in the cytosol [6], [8], [9].
3. Attenuation of the messenger protein's force of attraction toward the NM due to partial screening by other negatively charged proteins in the cytosol.

Our results demonstrate Coulomb interactions between the intracytoplasmic electric field and phosphorylated messenger proteins may play a critical and previously unrecognized role in cellular physiology.

## Methods

### Mathematical Models

Much of the environmental information takes the form of extracellular ligands (Fig 1) and is “measured” by the cell through cell membrane receptors that bind the ligands. Information is transmitted from the CM to the NM through one or more messenger proteins (Fig 1). Each messenger protein is typically “activated” by addition of a phosphate to a specific amino acid by a kinase that is also a messenger protein more proximal in the information pathway. Phosphorylation of the protein typically converts it to a kinase that phosphorylates the next protein in the sequence. Although the main function of phosphorylation is to alter the configuration and function of a protein, we note that it also adds *negative charge*. We propose that this negative charge is critical for information transmission from the CM to the NM because it allows the protein to interact with, and be accelerated by, an intracytoplasmic electric field (found next). Here we frame this hypothesis mathematically to understand the information dynamics that will result from these proposed interactions and compare these to the traditional model of simple diffusion.

Conditions of the model are:

1. The outer rim of the nuclear membrane is positively charged and generates an electric field in the cytoplasm. The field is not subject to shielding by inorganic ions such as K^+^ and Cl^−^ because they flow freely through the nuclear pores but is shielded by negatively charged proteins which can pass through the nuclear pore only via active transport.

2. The messenger proteins are free to diffuse [2] in the cytoplasm between the cell membrane and the nuclear membrane. The assumption of free diffusion is consistent with published observations [6], [8], [9].

3. We assume a typical mammalian cell with the parameters [6] in Table 1.

4. The valence *z* (or number of free electrons) of each messenger protein is allowed to vary as a function *z(t)* of the time due to phosphorylation
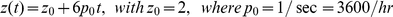
(1)is an average phosphorylation rate [10]. The factor 6 arises since phosphorylation of many messenger proteins adds 3 phosphates, or *3z_0_* = 6 negative charges, to the messenger protein. We assume that at/near the CM, the protein uptakes *z_0_ = 6* electron charges [10]–[12].

(5) Friction with the cytoplasm exerts a drag effect on each protein, where the drag coefficient *K* is a function [10]

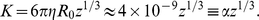
(2)This assumes each protein consist of a chain of 400–500 amino acids, which is the typical range of human messenger proteins. The resulting drag force is
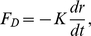
(3)with *dr/dt* the velocity.

**Table 1:** Parameters used in the simulations.

CM radius *r_0_*	5 micron
NM radius *a*	3 micron (Note: 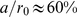 for mammalian cells)
Cytoplasm dielectric const.	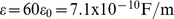
Thermal energy *kBT*	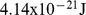
Positive charge on nucleus *Q_NM_*	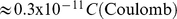
Viscosity *η* of cytoplasm	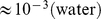
Reynolds number *R*_0_	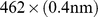

All values from Ref 10.

### RAF Experiment

To examine intracellular motion of messenger proteins we focused on the activation and movement of RAF which is a major component of the EGFR pathway (Fig 1) but also interacts with proteins in other pathways providing cross-talk. After a ligand binds to the EGFR complex, the signal is ultimately propagated in the membrane through GTP binding to RAS [12]. Activated RAS then “recruits” RAF to the cell membrane and adds multiple phosphates [13]. RAF then acts as a kinase for MEK within the EGFR pathway but also has the potential to interact with components of other pathways [14]. RAF is typically present in low concentrations (i.e. 0.0030 to 0.013 micrograms) [15]. RAF requires dephosphorylation of Ser259 suggesting that it is partially phosphorylated even in its basal, inactive state. Thus, RAF activation appears to first require a dephosphorylation prior to recruitment to the CM and addition of phosphates to a number of different sites [13]. The RAF protein has an estimated isoelectric point of about 9.0 but the isoelectric points after phosphorylation of different sites has not been measured. Based on our model, we hypothesize that coulomb interactions with the nuclear membrane *E* field, upon the addition of 6 phosphates (about 12 negative charges) [13], will rapidly result in movement of phosphorylated RAF toward the nuclear membrane. Our experimental strategy was to maintain the cells in media without serum to eliminate exposure to ligands so that the baseline state will have few messenger proteins in the cytoplasm and a largely unshielded E field. Upon addition of serum and the multiple associated ligands, we expect a very rapid transit of the initial cohort of phosphorylated RAF proteins. However, this burst of negatively charged proteins in the cytoplasm will tend to screen subsequent messenger proteins so that their movement will be slower and exhibit greater dispersal.

In the experiments 10^5^ MCF10T or MDA-mb-231 cells were seeded on coverslips. The former is a non-tumorigenic human breast cell line while the latter is a breast cancer cell line that is not known to have constitutive upregulation of RAF. The MCF10T cells incubated in DMEM:F12 without serum in 6 well plates overnight and the MDA-mb-231 were incubated in DMEM high glucose without serum. The normal serum used in the culture media (5% horse serum for the MCF10T cells and 10% fetal bovine serum for the MDA mb-231 cells) was added. The cells were fixed with 4% paraformaldehyede at four different time points: (1) prior to addition of serum, (2) immediately upon addition of serum (i.e. paraformaldehyde was mixed with the serum), (3) 30 seconds after addition of serum, and (4) 10 minutes after addition of serum.

The cells were fixed in 4% paraformaldehyde for 10 minutes and then incubated in 1%BSA / 10% normal goat serum / 0.3M glycine in 0.1% PBS-Tween for 1h to permeabilise the cells and block non-specific protein-protein interactions. The cells were incubated with phosphoRAF-1 antibodies (Abcam #ab1095) at a 1∶200 dilution for 12 hours at 37°C. The secondary antibody (red) was Alexa Fluor® 488 goat anti-rabbit IgG (H+L) used at a 1/1000 dilution for 1h . Wash 3× with 1× PBS for 5 minutes each wash. Next, incubate with the total RAF-1 (Abcam #ab78330) at a 1∶200 dilution for 12 hours at 37°C. Wash 3× with 1× PBS for 5 minutes each wash. Add secondary antibody against total RAF-1 which is conjugated to a green fluorescence probe (488nM). Incubate for 2 hours at 37°C. Wash 3× with 1× PBS for 5 minutes each wash. DAPI was used to stain the cell nuclei (blue) at a concentration of 1.43µM.

## Results

### Electric field strength within the cytoplasm

#### Theoretical Values

A major component of our hypothesis is the presence of an intracytoplasmic electric field. The field is due to *negatively charged* proteins in the cytoplasm and the *positive charge Q_NM_* on the NM (Table 1). The presence of charge on the NM has been measured in studies dating back several decades [16]–[18]. However, the role of the electric field generated by these properties has not been explored in part due to the assumption that the screening effects of the mobile ions in the intracellular fluid will result in complete screening of the membrane charges within 1 or 2 nanometers. We propose, however, that the nuclear membrane does not act as a typical charged surface in the Debye-Huckel model because it contains a large number of pores. The nuclear pores are well characterized and are permeable to small inorganic ions such as potassium and chloride but not to proteins. As a result, any excess of Cl^−^ ions that might collect around the positive charge of the outer layer of the membrane will dissipate due to diffusion along concentration gradients through the membrane. Mobile charged proteins on the other hand cannot flow through the pores (they require ATP-dependent active transport) and thus will screen the NM charge. In general, the field causes each protein, of negative charge *z(t)q*, to be attracted toward the nucleus and proteins with positive charge to be accelerated toward the CM.

**Figure 2:**
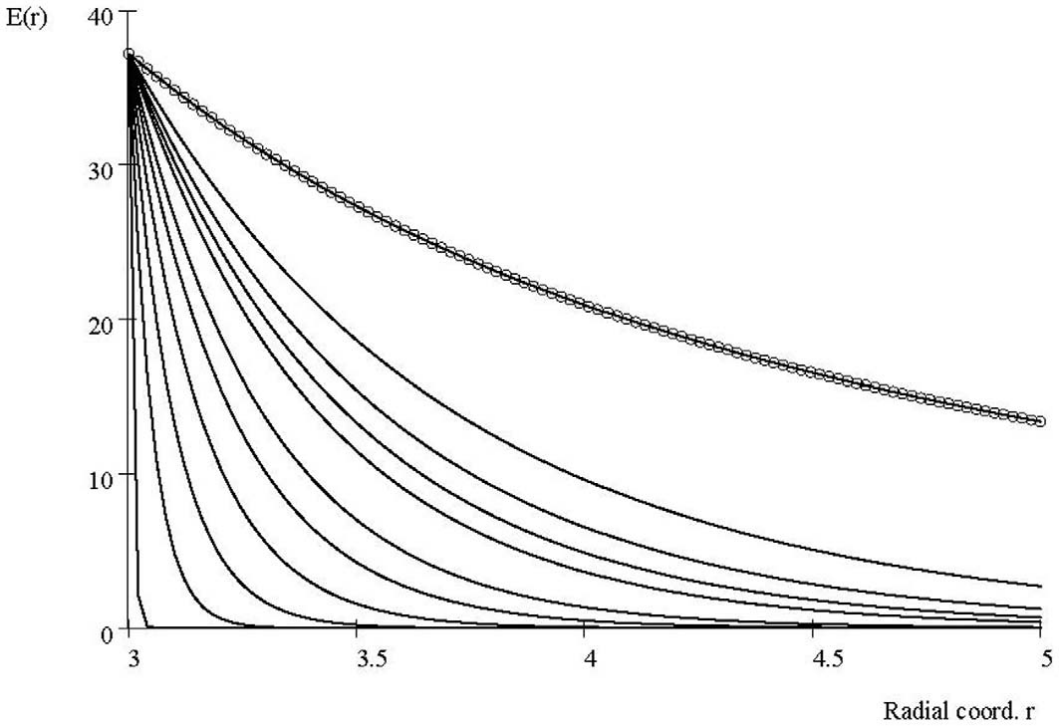
Calculated values of typical intracellular electric fields. Field strength *E* in units of 10^6^v/m for interaction parameter values plotted against distance *r* from the center of the cell in microns assuming the NM is at r = 3. The nuclear membrane is at r = 3. Each curve is for a different interaction parameter value 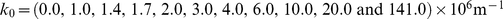. These correspond, respectively, to the presence of either a single protein 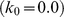; or clusters with a single class of proteins 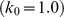 or 2 classes 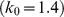, or 3, or…, or 100, or 400.

With 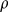 the total number/volume of these messenger proteins in the cytoplasm, the result is a *screened Coulomb law* of attraction to the nucleus,
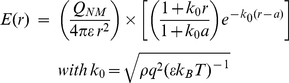
(4)

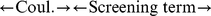
where *E(r)* is the force/charge or field strength at a distance *r* from the center of the cell. In the following, we will assume that nuclear membrane is at *r = a = 3µm*. By (4), the net force is the product of a Coulomb 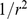 law with a screening term whose strength is governed by *k_0_*, the Debye-Huckel screening parameter. Some *E(r)* curves are plotted in Fig. 2 for characteristic values of *k_0_* based on the measured cytoplasmic concentrations of the mobile component of the EGFR pathway (RAF, MEK, and ERK) as calculated in Supporting Information. These demonstrate that *E(r)* decreases as the concentration of messenger proteins increases.

For comparison, the top (dotted) curve is the pure Coulomb 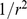 law obeyed by unscreened proteins. The curves below it show the degree to which the unscreened field is forced down by the screening factor through the Debye parameter *k_0_* . As *k_0_* increases there is an increase in the number of [9] proteins that simultaneously move through the cytoplasm (Appendix S1).

An exception is minimal screening at field points near the NM position 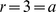. There all the curves approach a common value 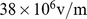. The reason is that, at such *r*, regardless of the protein density only a negligible number of proteins *can fit between* (and shield) the position *r* and the attracting charges on the NM. Conversely, once *r* is any significant distance from value 3, say at value 3.5, many ions can intervene, causing strong screening, and dependence upon parameter *k_0_* . Note that this theoretical value agrees well with experimentally determined values of 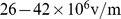 [14] based on measurement a NM transmembrane gradient of −13.3+/−0.1 mV in a Drosophila salivary cell with a nuclear diameter of 100–120µm. In Ref [12] the NM transmembrane gradient was −32+/−12 mV in human fibroblasts with NM diameter of about 15–20 µm. *k_0_* is defined by system parameters in the 2^nd^ Eq. (4), and in particular the protein number density value _. Each value of *k_0_* shown in Fig. 2 is found in Appendix S1 to result from a corresponding number of protein types moving in a cluster toward the nucleus.

For example, the value of 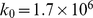, giving the 4th-highest curve, has the significance of holding for a scenario where three types of protein are moving together within the cytoplasm (see Appendix S1). Interestingly, this corresponds to recent observations suggest that RAF-MEK-ERK travel together in a chaperoned cluster that facilitates their interactions. Furthermore, as shown in Appendix S2, this value is in good agreement with estimates of the value of *k_0_* obtained from experimental mapping of the electric field discussed next.

#### Laboratory E Values

**Figure 3:**
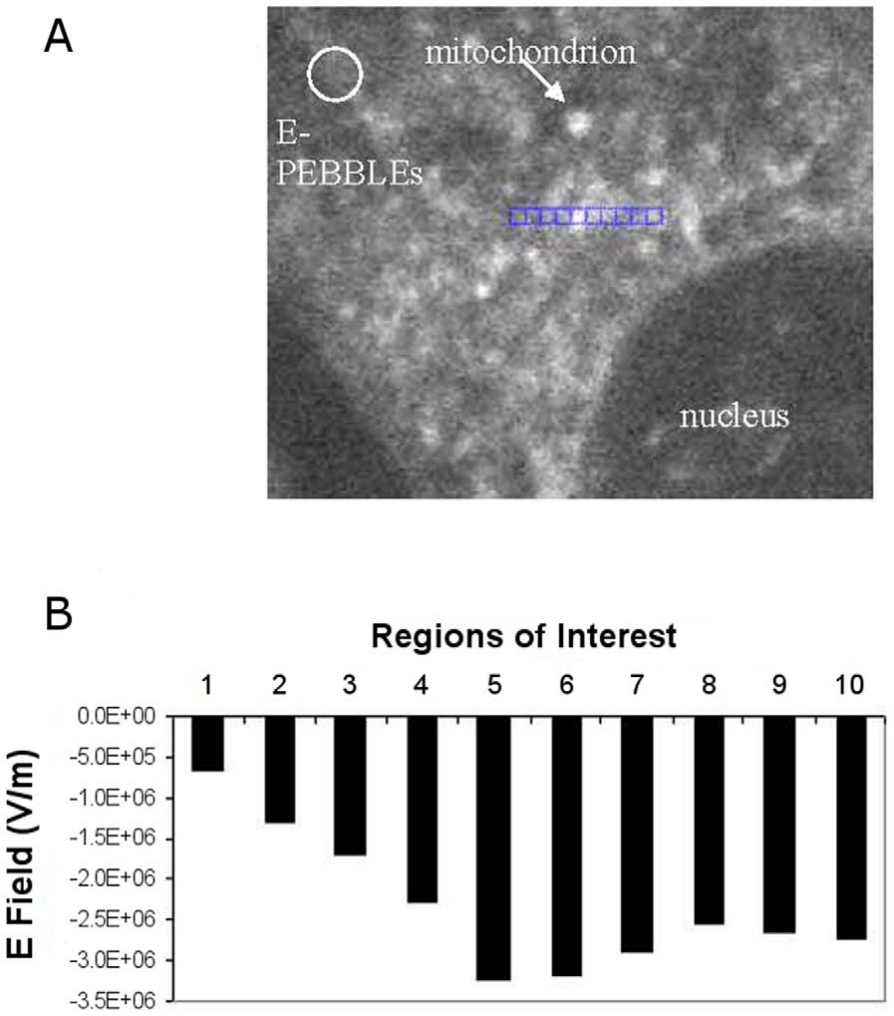
Measured values of intracellular electric fields using nanovoltmeters. Figure from Tyner, et al. (ref [20]). (A) *E* values shown as continuous tone intensity. (B) *E* values in the 10 boxes of A. Note that values of E generally decrease with distance from the NM. Regions 5 and 6 correspond to a bright spot similar to the mitochondrion shown higher in the image and so likely reflect the local influences of the charge in the mitochondrial membrane.

**Figure 4:**
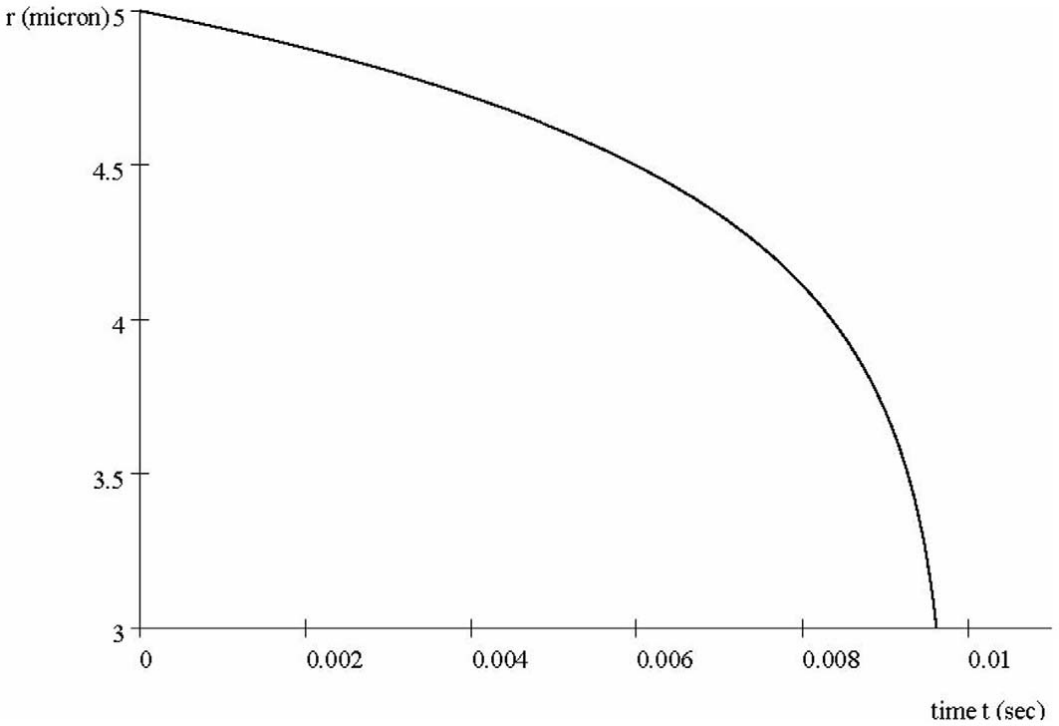
Transit of a typical phosphorylated protein cluster from the cell membrane to the nuclear membrane. Protein position *r* at time *t*, for a protein cluster with one phosphate group (2 electrons) and screening parameter 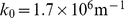. As discussed in Supporting Information, the latter defines a moving cloud consisting of 3 protein classes (i.e., RAF-MEK-ERK). The brief transit time 0.01s indicates a cloud *pulse*.

The accuracy of the model predictions and underlying assumptions can be tested against an experimentally determined map of the intra-cytoplasmic *E(r)* values that has recently been accomplished. These are shown as a continuous-tone intensity image in Fig. 3, from Tyner et al [20]. Using these date we can compare theory and laboratory results for *E(r)* values. With 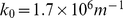 estimated by (see Appendix S2) and the cell parameters in Table 1, the screened Coulomb law (4) gives 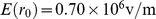 where r_0_ = 2microns. *r* can be estimated in Fig. 4 since the *ten* boxes of the figure span a length of *4.5*μm. Thus, *E(r_0_)* corresponds roughly to the value of former value in boxes 3 and 4 corresponding to measurement of 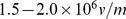. The value *E(a)* is the maximum value of *E* over all *r* that the theory (4) predicts and is not directly measured on Fig 3. However, the box closest to the NM measures about 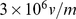 and is about 1 micron from the NM. In Fig 2, the predicted field is about 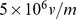. Of course, the nanovoltmeter readings are inferred from intensity readings, and errors of focus rapidly cause losses (errors) of intensity [16].

### Protein Trajectories

We next show results of using Eqs. (4)–(7) in Appendix S1. Fig. 4 shows the protein path *r(t)* for a protein with a two-electron charge 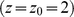, phosphorylation rate 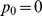, and in the presence of the Debye parameter value 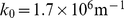. As shown, the protein transits from the CM at position 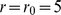 to the NM at 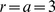 in a time 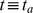 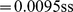 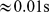. Also, the slopes represent velocities, giving an initial speed at 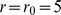 of about 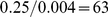
micron/sec, and at *r = 3* a speed of about 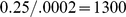
micron/sec. This is about a factor of 20 gain of speed, considerable despite the Coulomb shielding. The path *r(t)* for a phosphorylation rate 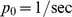 is about the same indicating that the rate of phosphorylation has little effect on the trajectory.

The dynamics of directed movement of messenger proteins can compared to the undirected, random diffusion which is often taken to be the mode of protein travel. It is instructive to compare the expected transit times from the two alternatives. The transit time 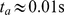 over a distance 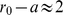 micron previously found (Fig. 4) for the directed motion amounts to an average velocity 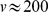 micron/s. We compare this 0.01s time with the time needed for the protein to *instead diffuse* through the cytoplasm in the usual root-mean square sense. The well-known [10] diffusion formula is
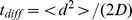
(5)where 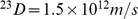 is the diffusion constant. This gives a root-mean square diffusion distance of 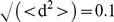
micron.

Hence, for undirected motion, in our (directed) transit time 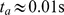, the protein cloud would move 0.1 micron. Furthermore, *this is in any direction*, e.g., toward the nucleus but also sideways and even back toward the CM. Thus, diffusion would result in broad dispersal of the messenger proteins throughout the cytoplasm and a wide range of transit time. This would result in minimal information transmission about the location and time at which a ligand arrived at the cell membrane.

#### Transit time compared to distance

**Figure 5:**
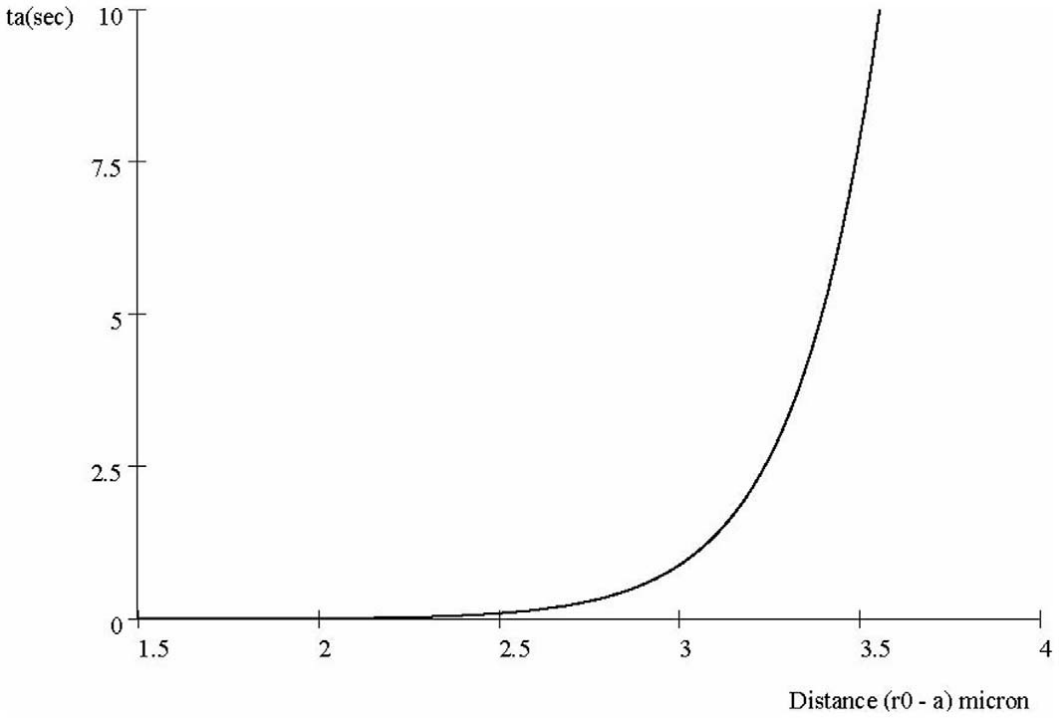
Transit time *t*_a_ for different cell sizes, with *p_0_* = 0.

Fig. 5 plots transit *t_a_* vs. distance from CM to NM. The curve shows explosive increase in *t_a_* once the distance exceeds about *3.0* micron. Fig 5 sets an upper limit to the distance between the nucleus and cytoplasm since it is likely that a NM more that about 3.5 microns from the CM will not allow the cell responds quickly to external signals. This represents a clear prediction of the model: a cell that need to process signals from the environment must locate its nucleus no more than about 3.5 microns from the region of the CM that harbors the relevant receptors.

#### Effect of Debye Parameter *k_0_*

**Figure 6:**
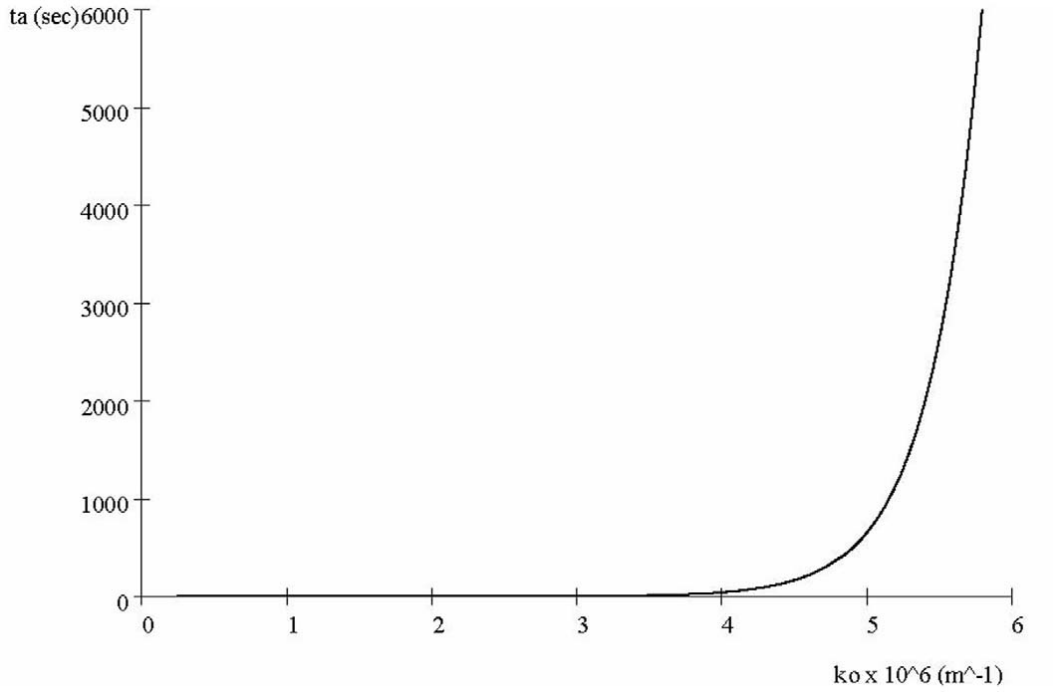
Effect of screening parameter *k*_0_ on transit time *t_a_*, with *p_0_* = 0.

We now ask how the screening parameter *k_0_* affects proteins transit. Since the shielding gets more severe as *k_0_* grows (Fig. 2), transit time *t_a_* should increase as *k_0_* increases. This conjecture is tested in Fig. 6 which shows transit times increase with *k_0_* with nearly a step dependence. The threshold for the step is at 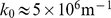. Although the screening effect is relatively minor up to this threshold, which correspond to low protein density values by the 2^nd^ Eq. (4), beyond it there is az very strong increase in transit time. As discussed below, the rapid increased in transit time as *k_0_* increases suggests a phase transition will occur as the number negatively charged messenger proteins increases (thus increasing *k_0_*). At low levels, transit time will be very fast but beyond a threshold value, transit time will increase significantly. The potential significance of this is discussed below.

#### Information flux F at the NM: message transit time vs. cytoplasm density

In the above analysis we have demonstrated threshold behavior in transit time based on the value of the screening parameter which is dependent on the density of negatively charged messenger proteins in the cytoplasm. How might this affect information processing by the nucleus? The criteria used by the nucleus to respond to information from the CM are not known. However, optimum processing of the information is likely dependent in some way on the intranuclear concentration of a messenger protein such as ERK which is the net result of influx and efflux. The former is dependent on *transit time t_a_ and density* 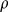 of the messengers. The latter is dependent on deactivation and removal of messenger proteins through nucleo-cytoplasmic shuttling.

We have discussed factors controlling transit time above. Messenger density 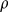 is likely dependent on the number of receptors activated at the CM and on amplification of that message by phosphorylation of multiple proteins with one or more pathways (cross talk). Note that these processes may be connected since rapid transit time will reduce opportunity for signal amplification and cross talk.

**Figure 7:**
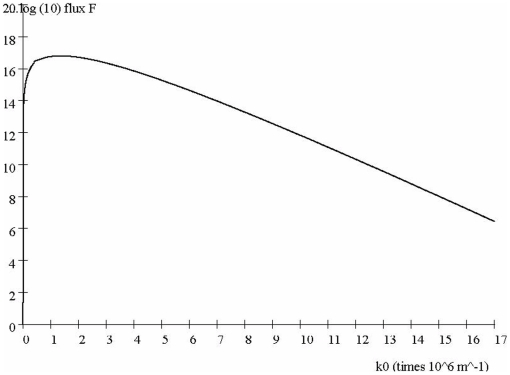
Flux *F* (proteins/area/ time) at the NM as a function of *k_0_*.

The relationship can be expressed as:
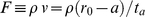
(6)The relationship of *F* to *t_a_* is demonstrated in Fig. 7 by Eq (6) and using the 2^nd^ Eq. (4) and Eqs. (B4)–(B7), is plotted vs. values of *k_0_*.

This confirms the step-like increase in transit time *t_a_* previously found in Fig. 7. In other words, in the absence of any system compensation, as *k_0_* increases transit time slows and messenger protein flux decreases.

However, the system dynamics are more complex. Consider a scenario in which *k_0_* is high and, by Fig. 7, transit time *t_a_* prolonged. A feedback effect may result because the increased *t_a_* allows the messenger proteins greater time in the cytoplasm allow them to activate more cytoplasmic messengers en route to the NM. This effectively increases their density 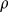 in Eq. (6), so increasing *F_max_* may be attainable over a wider range of Debye-Huckel parameters *k_0_* than in (7). Thus, Eq. (6) suggests two distinctly different system states may result in maximum information flux:

- a low *k_0_* state in which information received at the information from the CM is transmitted to the NM with maximal speed, or
- a high *k_0_* state in which messenger protein motion is slow, but allows for increased messenger density through signal expansion and inter-pathway cross talk.

In the case (i) of rapid protein movement, flux will be directly related to the activity of a single type of receptor. That is, the activation of a large number of receptors due to the sudden arrival of a wave of one type of ligand will initiate a rapid and specific response by the nucleus.

However, in case (ii) moderate numbers of different types of ligand are present, and these activate many different receptors. The result is the high *k_0_* - feedback effect mentioned above, except that here the message can more generally be amplified or reduced. Information from different pathways will enter the nucleus at different rates depending on these complex dynamics but will also be lost through nucleo-cytoplasmic shuttling. We speculated that a response at the nucleus will occur only when the net concentrations of different types of messenger proteins, determined by the addition of messengers through CM-NM transit and subtraction through nucleo-cytoplasmic shuttling [8], exceed certain thresholds. These considerations effectively replace the bare *arrival* rate *F* by an overall information *processing* rate in bits/sec.

Thus, a requirement of a maximum information rate of proteins at the NM implies that proteins from a single pathway travel together in a pulsed cloud, causing the largest number of proteins per unit area to reach the NM in the minimum amount of time. These particle rate conditions permit optimal processing, e.g. by simple majority decision-rule [21] (see above) among proteins of the same type, so that the highest level of error rejection is achieved. As we saw, this was in the minimum amount of time as well and a highest protein flux rate *F_max_*. Indeed, experiments in protein signaling [16] have suggested that nucleo-cytoplasmic shuttling may be sufficiently fast to keep up with such a high rate of arrival of proteins at the nucleus. The authors [21] describe this as “a filter for high frequency signaling in the cytoplasm.”

Taken together, *these properties accomplish the highest possible bit rate for the system*– the Shannon information capacity of the cell channel [22].

### Intracellular RAF movement

Because of the high speed of protein movement predicted, this will be difficult to measure with current experimental methods. Burack et.. al. [23] using live cell imaging have demonstrated extremely rapid movement of ERK into the nucleus in serum-starved cells following addition of EGF to the culture media (all labeled ERK entered the nucleus in less than 60s after a lag phase with no movement of 60s thought to be due to ligand binding and signal processing in the CM).

We attempted to examine movement of RAF which is upstream of ERK and should be phosphorylated more rapidly after EGFR binding. Furthermore, while the location of ERK within the cytoplasm is not known, it is clear that RAF will be phosphorylated at the cell membrane so that this must be the starting point of its transit to the nucleus. Furthermore, unphosphorylated RAF has a pKa of about 9.2 so that it will be positively charged at the normal intracellular pH of about 7.3. We note that RAF is maintained in a partially phosphorylated state which will add negative charge possible resulting in a near neutral state at baseline. However, upon addition of ligand, RAF is initially dephosphorylated [13] resulting in a strong positive charge that, upon interacting with the cytoplasmic *E* field, will accelerate it rapidly toward the cell membrane.

**Figure 8:**
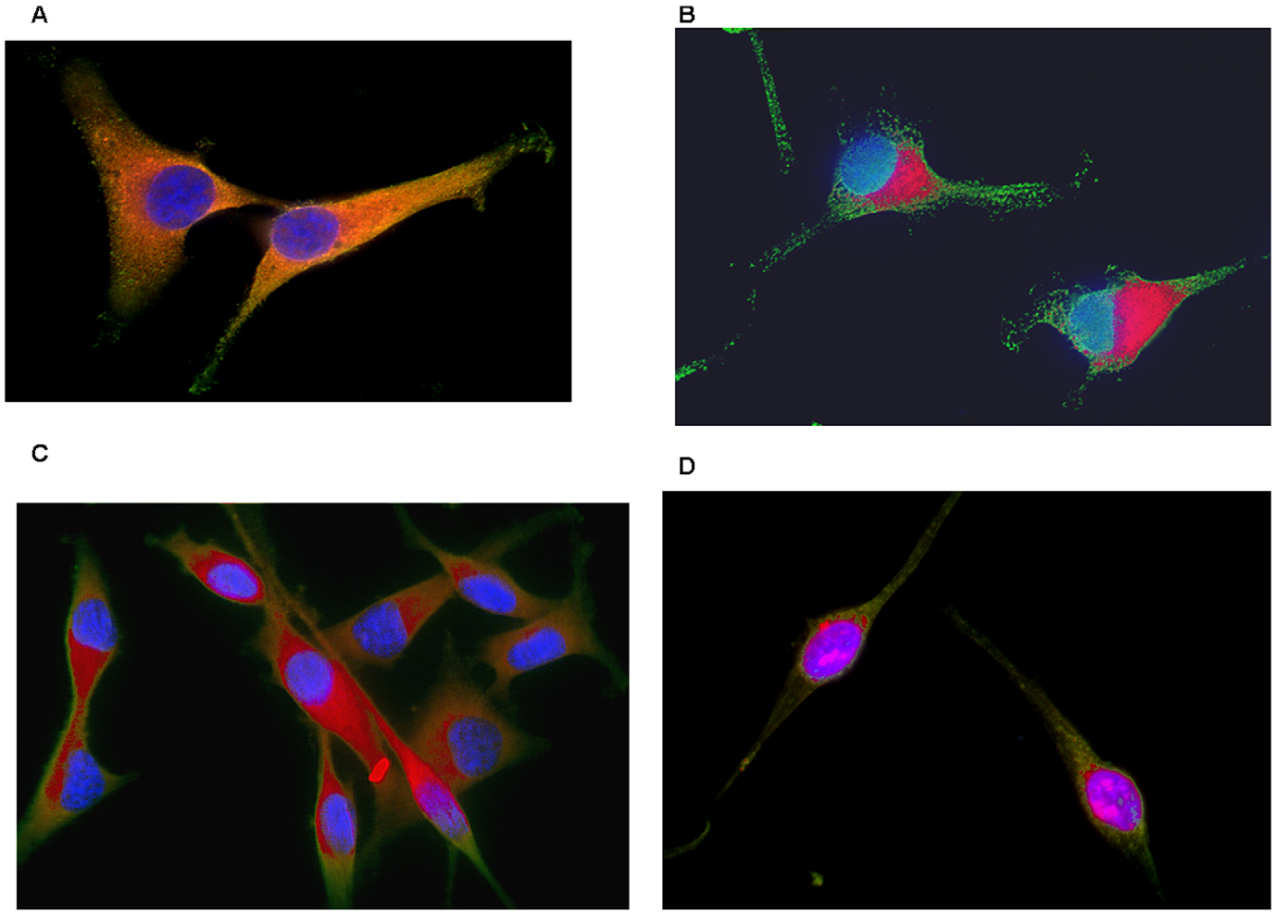
Distribution of pRAF and total RAF at various times following addition of serum to cells that had previously been serum-starved. MDA-mb-231 cells stained for pRAF (red) and total RAF (green). The nucleus is stained with DAPI (blue). 8A. Cells after 12 hours of culture without serum with diffuse intracytoplasmic distribution of RAF. This is consistent with an expected neutral charge (RAF has a pKa of 9.2 but unactivated RAF is partially phosphorylated [13]) 8B. Cells in which serum and fixative were added simultaneously. Despite this extremely short interval between stimulation and fixation, pRAF is asymmetrically clustered around the nuclear membrane 8C. Cells fixed 30 seconds after addition of serum, pRAF again is clustered around the nucleus but with more symmetric distribution. 8D. 10 minutes after serum, pRAF is tightly packed in the nuclear membrane.

Representative observations from multiple experiments with both cell lines are presented in Fig 8. After incubation without serum, both cell lines demonstrated diffuse distribution of RAF throughout the cytoplasm (Fig 8A). This is consistent with published observations in other cell lines. Immediately following addition of serum, pRAF was observed asymmetrically distributed around the nuclear membrane (Fig 8B) while the remaining unphosphorlated RAF proteins clustered around the cell membrane. This is consistent extremely rapid signal transduction that will require multiple steps including: dephosphorylation of RAF at the SER259 site, movement of RAF to the CM, phosphorylation of multiple activating sites, and movement of phosphorylated RAF to the NM. Since the fixative was added simultaneously with the ligands, all of the RAF proteins exhibited rapid movement, consistent with the hypothesis of directed rather than random walk motion. The asymmetric distribution of pRAF around the nucleus is probably related to variations in the EGFR sites in the CM. At 30 seconds Fig. 8C), pRAF was more diffusely distributed with the cytoplasm around the NM as expected with higher levels of shielding. At 10 minute (Fig 8D) all of the pRAF is clustered tightly around the NM.

**Figure 9:**
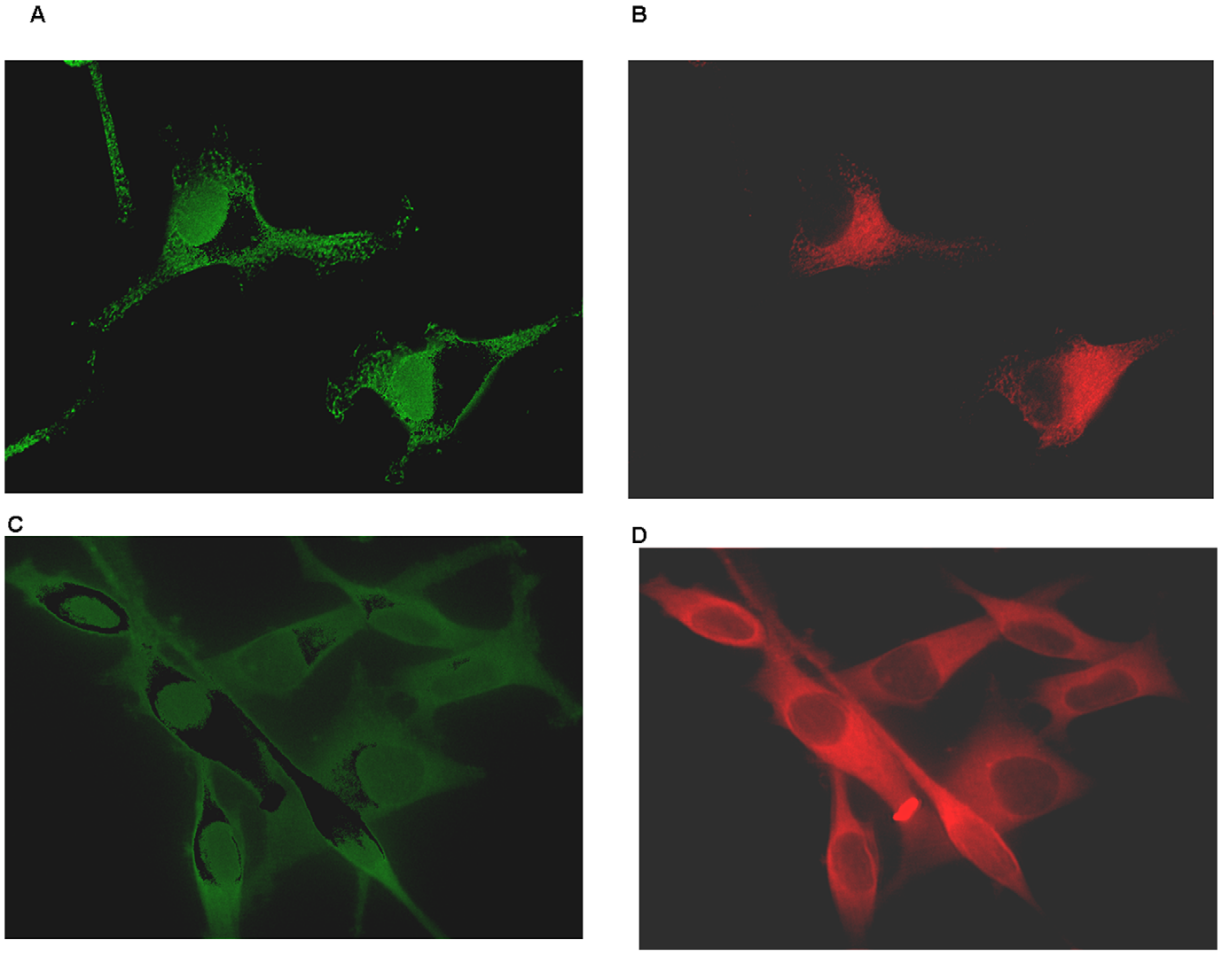
Distribution of pRAF and unphosphorylated RAF at various time following addition of serum to previously serum starved cells. Subtraction images from the cells from Fig 8 demonstrate the separation of pRAF from unphosphrylated RAF. 9A and B demonstrate the perinuclear localization of pRAF and the peri-cytoplasmic distribution of unphosphorylated RAF in cells immediately following additions of serum. 9C and D show the distribution 30 seconds after serum addition.

To better demonstrate the regional variations in pRAF and RAF, color subtraction images from Fig. 8 are shown in Fig 9. The top row are images from the time serum is added showing pRAF clustered around the nucleus and unphosphorylated RAF confined to the region of the CM. The lower row shows images 30 seconds after addition of serum and show a similar pattern but with broader distribution of pRAF.

## Discussion

Normal cell function requires timely and accurate transmission of information from receptors on the cell membrane (CM) to the nucleus. Movement of messenger proteins in the cytoplasm is thought to be dependent on random walk. However, we note that Brownian motion will disperse messenger proteins throughout the cytosol resulting in slow and highly variable transit times. We propose a new model of intracellular information flow in which movement of negatively charged phosphorylated messenger proteins is *directed* by coulomb interactions with an intracellular electric field toward the positively charged nucleus. We use published date on the transmembrane potential on the nuclear membrane to calculate the characteristics of the field that are consistent with recent measurements using nano-voltmeters. We demonstrate this field will accelerate negatively charged, phosphorylated messenger proteins toward the nucleus while random walk dispersed proteins throughout the cytoplasm resulting in a transit time that can vary widely among a cohort of messengers simultaneously leaving the CM.

We also demonstrate that the movement of the proteins can be mitigated by screening that results from a large number of charged proteins travelling in the cytoplasm. This occurs, for example, when multiple ligands are continuously binding to receptors on the cell membrane. This will result in two general patterns of information flow:

First, when a low-density, quick pulse of ligands arrives at the cell membrane, the information is transferred very rapidly (transit time of less than 1 second) to the nucleus for immediate action. In this scenario, there is essentially no time to allow cross-talk among the pathway. Information transfer takes the form of a narrow, pulsed cloud of proteins. Despite the low density of these proteins, their speed is sufficiently great to deliver an optimally high rate of information to the nucleus.

Second, chronic information integration for cell maintenance can also be attained through the activation of moderate numbers of different types of ligand receptors. In this case, the chronic low level of messenger proteins increases the screening and transit time. Contrary to the first scenario, this setting with longer transit times increases cytoplasmic dwell times allowing greater signal expansion and cross-talk among the pathways. The result is a high *k_0_* - feedback effect where, owing to increased transit times *t_a_*, the message can generally be amplified and integrated with information flowing in other pathways.

A major assumption of our model – that the positively charged outer surface of the NM will result in an *E* field extending a few microns into the cytoplasm - represents a significant variation from traditional models. Application of the Debye-Huckel model to the cell membrane assume that shielding by mobile intracellular inorganic ions (primarily K^+^ and Cl^−^) will result in an E field extending only 1 or 2 nanometers from the membrane. We propose that, because inorganic ions are freely permeable through the NM as a result of the properties of nuclear pores, they do not in fact shield the NM charge. This relies on the assumptions that the time necessary to pass through an NM pore is small compared to the time to thermodynamic equilibrium of the cytoplasm. For an ion to speedily get through an NM pore, it should be narrower in diameter than the pore diameter. A Cl^−^ ion has a diameter of 181 pm (picometer), K^+^ ion of 138 pm [24]. By comparison, average pore diameter in NM = 90 nm [25]. This is 500 times that of the Cl^−^ ion so that multiple ions could pass through the pore simultaneously. Finally, the relaxation time to thermal equilibrium in liver cells measured experimentally ranges from about 0.1 to 1microseconds [26]. By comparison, RNA molecules, which are much larger than the ions but do possess charge, pass through 1.5 nm wide pores of carbon nanotube membranes in 10 ns (nanosecond) [27]. This is one thousandth, or less, of the above equilibrium time for liver cells. This obeys our time requirement. Thus, while there are no experimental data that has explicitly measured the transit times through NM pores of Cl^−^ and K^+^ ions, indications from other, related experiments is that they pass through the NM pores much faster than the time to reach thermal equilibrium in the cell, so that the Debye-Huckel field is unaffected by them.. Interestingly, we note that there is thought to be some cellular control of nuclear pore patency. If so, cells could effectively turn the intracytoplasmic *E* field on and off by opening and closing the nuclear pores respectively as the latter would reduce the Debye length to about 1 nanometer.

Thus, we propose that inorganic ions do not contribute to screening of the intracellular electric field nor do most organic anions in the cell, which are fixed. However, the *E* field is shielded by messenger proteins that are both mobile in the cytosol and unable to move quickly through the nuclear pores (i.e. they require active transport). The validity of our assumptions is supported by results that predict values of the E field within the cytoplasm that are consistent with recent measurements. Furthermore, the experiments performed as part of this study, while limited, also support the modeling results.

Clearly, confirmation of this theoretical approach will require far more extensive model development and experimental observations. However, we propose that the model of directed messenger protein movement due to Coulomb interactions with an intracellular electric field is both plausible and a reasonable alternative to the standard model of diffusive protein motion.

## Supporting Information

Appendix S1 (0.07 MB DOC)

Appendix S2 (0.04 MB DOC)
