# Supplementary material for: Coulomb Interactions between Cytoplasmic Electric Fields and Phosphorylated Messenger Proteins Optimize Information Flow in Cells
Source: PLoS One. 2010 Aug 11;5(8):e12084. doi: 10.1371/journal.pone.0012084 (PMC2920310; doi:10.1371/journal.pone.0012084)
Supplement: Appendix S1 — (0.07 MB DOC) [file pone.0012084.s001.doc]

**APPENDIX S1**

*Terminal velocity approximation*

By Newton’s 2^nd^ law the net force on a protein of mass *m* is *F = m(d ^2^r/dt^2^) = F_EM_ + F_D_*, where *F_EM_ = - z(t)qE(r)* is the electrostatic force of attraction to the NM and *F_D_* = *-Kdr/dt* is the drag force (3) due to friction with the cytoplasm. Combining gives

$-dr/dt=(m/K)(d^{2}r/dt^{2})+z(t)qE(r)/K$ $\equiv T_{1}+T_{2},$ $m=50$kDa$\approx10^{-21}$kg. (B1)

We next show that under reasonable conditions the acceleration term $T_{1}<<T_{2},$the charge *q*-dependent term.

The given value (B1) for *m* and (2) for *K* give *m/K*=$0.2\times10^{-12}.$ Hence $T_{1}=0.2\times10^{-12}(d^{2}r/dt^{2}).$ Also, by (1), *z(t)_min_* = 2, by Fig. 3C *E(r)_min_* $\approx0.5\times10^{6}$, the electron charge *q*$=1.6\times10^{-19}$Coul., and $K\approx5\times10^{-9}$ from (2). These give *T_2min_ = $0.32\times10^{-4}$.*  Then $T_{1}/T_{2 \min}=0.6\times10^{-8}(d^{2}r/dt^{2})$. Hence $T_{1}<<T_{2}$ unless the acceleration $d^{2}r/dt^{2}\geq1.7\times10^{8}$m/s^2^ . Such a high acceleration requires a change in velocity, e.g., of $1.7\times10^{5}$m/s within but 1 millisec. But such high speeds and accelerations are unreasonable within the cytoplasm of a cell. Therefore $T_{1}<<T_{2},$ so that to an extremely good approximation, (B1) becomes

$z(t)qE(r)+Kdr/dt=0,$ with $r(0)=r_{0}$ (2)

a boundary condition. Velocity *dr/dt* is typically called the 'terminal velocity' of the motion, although it effectively holds at all times in this approximation. Next, using the form (4) for *E(r),* and dividing by $z^{1/3}$, the dynamics obey

$\frac{z^{2/3}(t)qQ_{NM}}{4\pi\varepsilon r^{2}}\left(\frac{1+k_{0}r}{1+k_{0}a}\right)e^{-k_{0}(r-a)}+\alpha dr/dt=0, \alpha\equiv K/z^{1/3}.$ (3)

This is merely a *first-order*, *linear* differential equation.

*Solutions*

1. *Case of* $p_{0}\neq0$

The differential equation may be analytically integrated, giving a solution $t=\frac{1}{2p_{0}}[3^{-3/5}(10p_{0}B(r)+3z_{0}^{5/3})^{3/5}-z_{0}]$, with (4)

$B(r)=C\left[e^{1+k_{0}r_{0}}(k_{0}r_{0}-2)-e^{1+k_{0}r}(k_{0}r-2)+Ei(1+k_{0}r_{0})-Ei(1+k_{0}r)\right]$ and (5)

$C=\frac{4\pi\alpha\varepsilon}{eqk_{0}^{3}Q_{NM}}\left(\frac{1+k_{0}a}{\exp(k_{0}a)}\right)$. (6)

Also, *Ei* is the exponential integral function
