# Supplementary material for: Coulomb Interactions between Cytoplasmic Electric Fields and Phosphorylated Messenger Proteins Optimize Information Flow in Cells
Source: PLoS One. 2010 Aug 11;5(8):e12084. doi: 10.1371/journal.pone.0012084 (PMC2920310; doi:10.1371/journal.pone.0012084)
Supplement: Appendix S2 — (0.04 MB DOC) [file pone.0012084.s002.doc]

**APPENDIX S2**

*Parameter k_0_ from Experimental E-Data*

Figs. 3B and C allow a crude *laboratory* estimate of *k_0_* to be made. First, the caption in Fig. 4 states that *ten* blue-colored boxes of the figure span a length of *4.5μ*m in the cell. Thus each cell spans *0.45*μm. Also, the total length of the 10 boxes on the page is measured as about *14.5* mm. Then *4.5*μm/*14.5*mm = *0.31*μm length in the cell corresponds to 1 mm on the page.

We'll also need to know the effective pixel size of the imaging. Each image pixel value shows the local *E* value over the extent of a "nanosized voltmeter" called an E-PEBBLE. This is physically a silane-capped micelle $\approx30$nm = 0.03μm in diameter containing a fluorescent, voltage-sensitive dye. Hence 30 nm is likewise the effective pixel size when the plane of the cell is imaged in focus.

*Biological Significance of Values k_0_*

The value of *k_0_* for a given cell scenario is defined by system parameters in (4), and in particular the protein number density value **. The latter is computed on the following basis.^13^

Consider a typical mammalian (liver) cell of volume 3.4×10⁻¹⁵m³. (The authors assume a cubic shape, but the resulting computed values of ** and *k_0_* should be independent of any assumed shape.) There are roughly 7.9×10⁹ protein molecules per mammalian (liver) cell including proteins of all types. Also, there is a range of from 20,000 to 1,000,000 different types of protein in the cell: examples of such types are RAF, MEK, or ERK . We arbitrarily choose the figure 20,000, noting that higher choices will lead to smaller characteristic values of *k_0_* , and therefore larger interaction lengths *1/k_0_* than found below. On this basis there are (7.9×10⁹)/20000 = 3.95×10^5^ protein molecules of any *one kind* in the cell.

These define *two* classes of densities. The density $\rho_{ac}$includes *all 20,000 classes* of protein, and has value$\rho_{ac}$ = (7.9×10⁹)/(3.4×10⁻¹⁵) = 2.3×10²⁴m⁻³. Or, the density $\rho_{1c}$ of any one class of protein is 1/20,000 of the value of $\rho_{ac}$, or $\rho_{1c}$ = 1.15×10²⁰m⁻³.

Finally we use these, and the constants listed in Table 1, in the 2^nd^ Eq. (4) to get two classes of parameter values *k_0_* ,

(*k*_0_)_1c_= 1.00×10⁶m^-1^ and (*k*_0_)_ac_ =141.0×10⁶m⁻¹. (1)

The first figure (by the way, not exactly 1 to higher decimal places) is for one protein class, such as RAS, the second for all 20,000 classes assumed. By the 2^nd^ Eq. (4), *k_0_* increases as the *square root of the number of classes* of protein in the cell (assuming, for simplicity, that each class occurs equally). Then respective figures *k_0_* for the simultaneous presence of either 1 (say RAS), or 2 (say RAS and RAF), or 3 (RAS, RAF and MEK), 4 (RAS, RAF, MEK and ERK), or 9, 16, 36, 100, 400, or all possible protein classes, are

*k_0_* = 1.0, 1.4, 1.7, 2.0, 3.0, 4.0, 6.0, 10, 20, and 141.0*×10*^6^m⁻¹. (2)

Resulting field strength curves *E(r)* using (4) are plotted in Fig. 2.
